# Supplementary material for: IL-33 and MRGPRX2-Triggered Activation of Human Skin Mast Cells—Elimination of Receptor Expression on Chronic Exposure, but Reinforced Degranulation on Acute Priming
Source: Cells. 2019 Apr 11;8(4):341. doi: 10.3390/cells8040341 (PMC6523246; doi:10.3390/cells8040341)
Supplement: Supplementary file 1 [file cells-08-00341-s001.pdf]

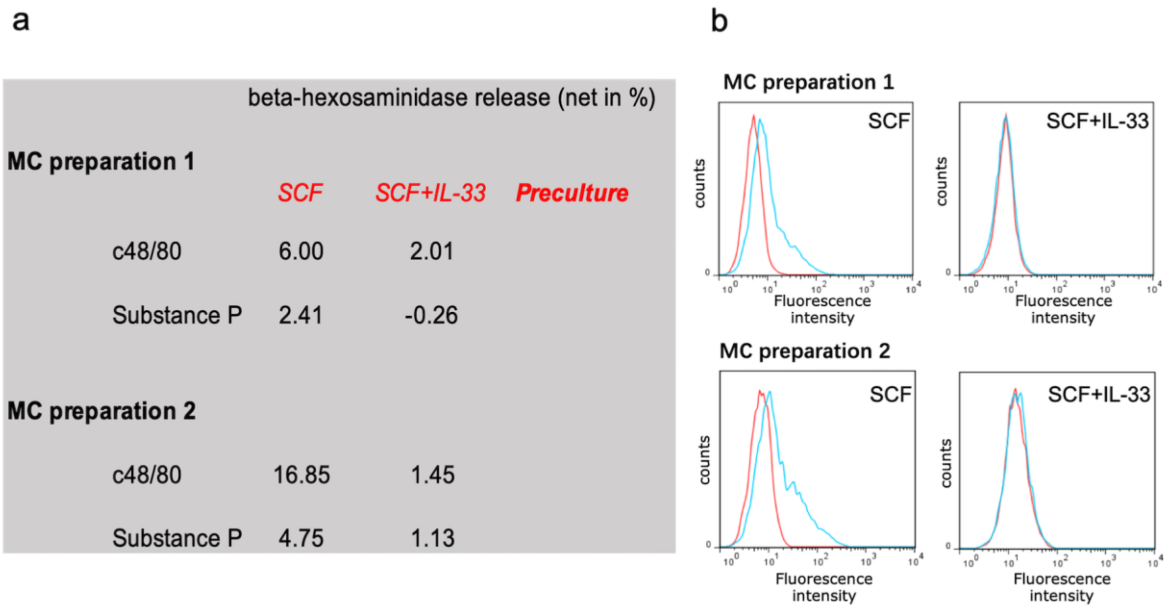

MC preparation 1	SCF	SCF+IL-33	Preculture
c48/80	6.00	2.01	
Substance P	2.41	-0.26	
**MC preparation 2**			
c48/80	16.85	1.45	
Substance P	4.75	1.13	

Figure S1. Foreskin MCs downregulate the MRGPRX2 route upon long-term IL-33 treatment. Cells of two individual preparations were cultured in SCF only or SCF and IL-33 for five weeks, after which time cell were stimulated by c48/80 or SP (Substance P) and MRGPRX2 surface expression quantified, as in Figure 1. (**a**) Net beta-hexosaminidase release elicited by C48/80 and SP, respectively, note that the release is very low in MC preparation 1, which was also occasionally the case in Figure 1a. This is related to SCF's negative effect on MRGPRX2 expression and function, as described in Reference [19]. (**b**) MRGPRX2 cell surface expression, as determined by flow-cytometry, histograms for the two independent foreskin MC cultures are depicted, red: Isotype, blue: MRGPRX2-specific antibody.

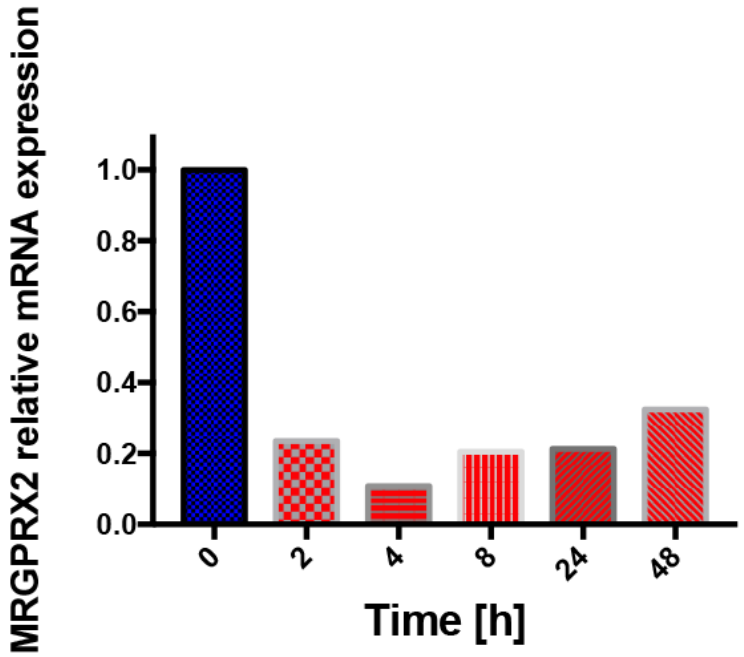

Figure S2. Time course of MRGPRX2 mRNA expression upon IL-33 treatment. Cells were deprived of growth factors overnight and treated with IL-33 (20 ng/mL) for the indicated times. Control cells were kept in medium only and both untreated and IL-33 treated cells were harvested at each time

point. MRGPRX2 expression in the presence of IL-33 was normalized to the matching non-treated control for each time point. One experiment out of two is shown.

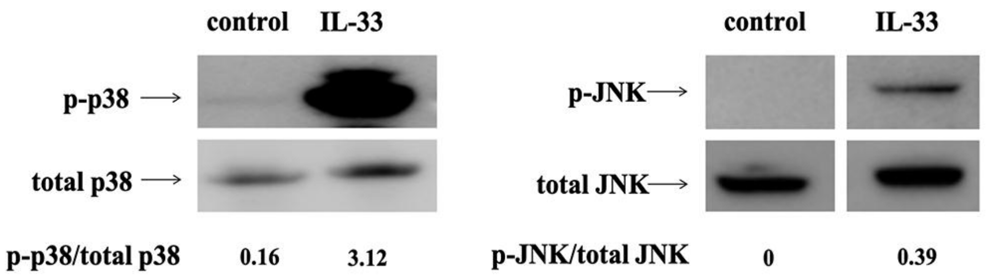

Figure S3. JNK and p38 are phosphorylated upon IL-33 administration. MCs were deprived of growth factors overnight, then stimulated with IL-33 (20 ng/mL) for 15 min. Representative immunoblots for p-JNK and p-p38 are depicted and reproduce the findings recently published by us [24]. Blots were stripped and re-probed with antibodies against total JNK and p38. The bands were quantified by densitometry and the ratios of phosphorylated/total protein are given in the figure.

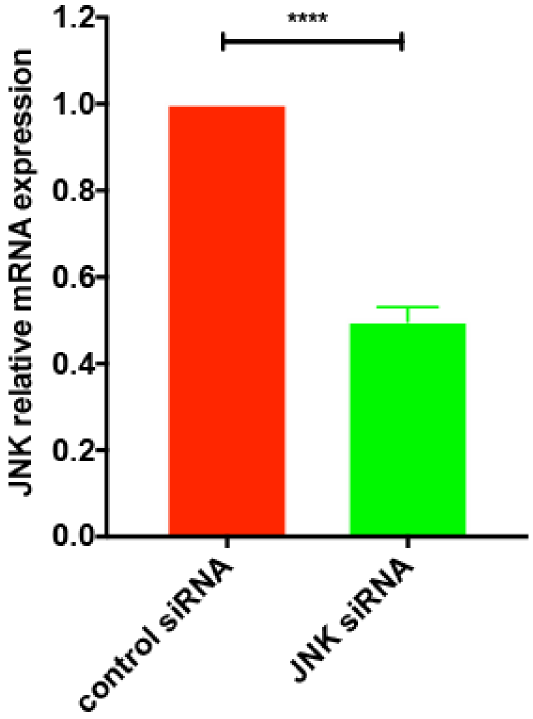

Figure S4. JNK knockdown (KD) efficiency. MCs were treated with JNK1-specific siRNA or control non-target siRNA, as described in Materials and Methods. Knockdown efficiency was determined by detecting JNK1 mRNA expression by RT-qPCR. Data are the mean ± SEM of $n = 5$ experiments. **** $p < 0.0001$.

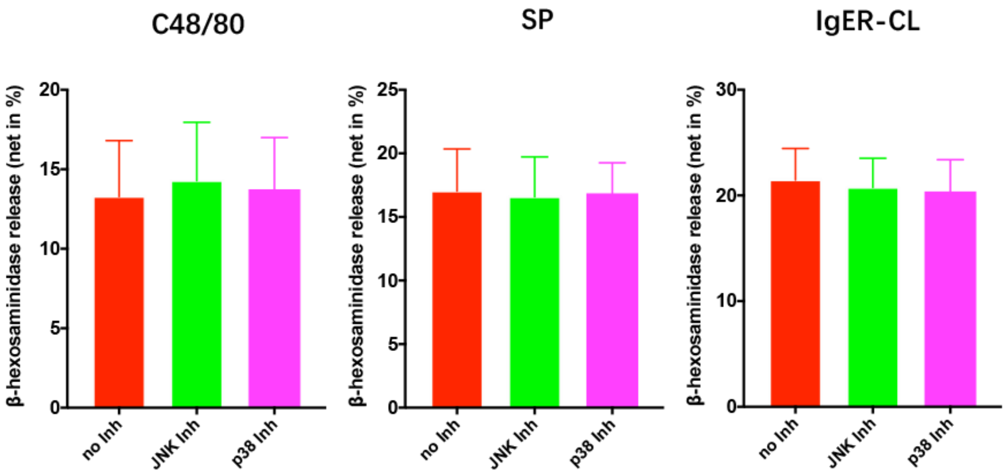

Figure S5. MC degranulation in the absence of IL-33 priming does not depend on either JNK or p38 activity. Cells were pretreated with the inhibitors SB203580 (p38) or SP600125 (JNK) as in Figure 5, but omitting the addition of IL-33, then stimulated by C48/80 (10 μg/mL), SP (30 μmol/L), or FcεRI aggregation (AER-37, 0.1 μg/mL). The release of β-hexosaminidase was determined. Data are presented as net β-hexosaminidase in percent of total β-hexosaminidase (mean ± SEM, $n = 7$). Inh = inhibitor, CL = cross-linking.
